# Increased Frequency of Circulating Follicular Helper T Cells in Patients with Rheumatoid Arthritis

**DOI:** 10.1155/2012/827480

**Published:** 2012-05-10

**Authors:** Jie Ma, Chenlu Zhu, Bin Ma, Jie Tian, Samuel Essien Baidoo, Chaoming Mao, Wei Wu, Jianguo Chen, Jia Tong, Min Yang, Zhijun Jiao, Huaxi Xu, Liwei Lu, Shengjun Wang

**Affiliations:** ^1^Department of Laboratory Medicine, The Affiliated People's Hospital, and School of Medical Science and Laboratory Medicine, Jiangsu University, Zhenjiang 212013, China; ^2^Department of Nuclear Medicine, Affiliated Hospital of Jiangsu University, Zhenjiang, China; ^3^Department of Pathology and Center of Infection and Immunology, The University of Hong Kong, Hong Kong; ^4^Zhenjiang Key Laboratory of Medical Immunology, Affiliated Hospital of Jiangsu University, Zhenjiang, China

## Abstract

Follicular helper T (Tfh) cells are recognized as a distinct CD4^+^ helper T-cell subset, which provides for B-cell activation and production of specific antibody responses, and play a critical role in the development of autoimmune disease. So far, only one study investigated the circulating Tfh cells increased in a subset of SLE patients. Since relatively little is known about the Tfh cells in rheumatoid arthritis (RA) patients, in this study, Tfh-cell frequency, related cytokine IL-21, and transcription factor Bcl-6 were investigated in 53 patients with RA and 31 health controls. Firstly, we found that the frequency of CD4^+^CXCR5^+^ICOS^high^ Tfh cells was increased significantly in the peripheral blood of RA patients, compared with that in healthy controls. It is known that Tfh cells are critical for directing the development of an antibody response by germinal centers B cells; secondly, we observed that the Tfh-cell frequency is accompanied by the level of anti-CCP antibody in RA patients. Furthermore, expression of Bcl-6 mRNA and plasma IL-21 concentrations in RA patients was increased. Taken together, these findings have shown that the increased frequency of circulating Tfh cells is correlated with elevated levels of anti-CCP antibody, indicating the possible involvement of Tfh cells in the disease progression of RA.

## 1. Introduction

Rheumatoid arthritis (RA) is a chronic and symmetric polyarticular arthritis that primarily affects the small diarthrodial joints of the hands and feet [1]. The salient features of RA include the presence of circulating autoantibodies, dysregulated lymphocyte activation, and linkage to MHC class II [1]. Although both T cells and B cells are involved in the disease pathogenesis, CD4^+^ T cells and their cytokines are thought to play a crucial role in the induction and propagation of the inflammatory conditions. With the help of T cells, activated B cells migrate into lymphoid follicles of lymphoid organs and form germinal centers (GCs) [2]. Within the unique milieu of the GCs, follicular B cells undergo somatic hypermutation and affinity maturation, resulting in the diversification and selection of B-cell repertoire for and differentiate into antibody-secreting plasma cells and memory B-cell [3, 4]. Current studies have indicated a fundamental function of CD4^+^ T cells in regulating B cells proliferation and antibody production especially in the GC structures [5].

Recently, follicular helper T (Tfh) cells, a novel CD4^+^ T subset, have been found to be present in GCs [6], which regulate the development of antigen-specific B-cell immunity *in vivo* [7]. Tfh cells provide selection signals to GCs B cells and play an essential role in mediating long-lived antibody responses. The phenotypic and functional features of Tfh cells include surface expression of the chemokine receptor CXCR5 [chemokine(C-X-C motif) receptor 5], IL-21, and B-cell CLL lymphoma-6 (Bcl-6) [8, 9]. High levels of CXCR5 expression facilitate the homing of Tfh cells to B-cell follicles whereas Bcl-6 is essential for the generation of Tfh cells and functions in a gene dose-dependent manner [10]. It becomes clear that IL-21 produced by Tfh cells serve as an important regulator of humoral responses by directly regulating B-cell proliferation and class switching [5]. However, little is currently known about the potential role of Tfh cells in autoimmune pathogenesis.

An elegant study by Simpson et al. [11] has recently shown that the frequency of circulating CD4^+^CXCR5^+^ICOS^high^ Tfh cells was increased in SLE patients, which prompted us to examine the frequency of circulating Tfh cells in the peripheral blood of RA patients and its correlation with autoantibody production. In this study, the increased frequency of CD4^+^CXCR5^+^ICOS^high^ circulating Tfh cells was detected in RA patients, which was positively correlated with high levels of serum anti-CCP antibody. Thus, these results have indicated the possible involvement of Tfh cells in the pathogenesis of RA.

## 2. Materials and Methods

### 2.1. Patients

A total of 53 RA patients and 31 health controls were enrolled in the present study. Fifty-three newly diagnosed RA patients without treatment from 2009 to 2010 at the Affiliated People's Hospital of Jiangsu University were included in this study. RA patients fulfilled the 1987 revised criteria of the American College of Rheumatology (ACR) [12]. Thirty-one healthy volunteers were recruited as controls. Peripheral blood samples were obtained from all patients and healthy controls. The clinical characteristics were collected at the same time points as the plasma samples. Data describing the study subjects are summarized in Table 1. Ethical approval was obtained from Jiangsu University, and written informed consent was obtained from all individuals.

### 2.2. Cell Isolation

Plasma was collected through centrifugation and stored at −80°C for measurement of cytokine levels. PBMCs were isolated by standard Ficoll-Paque Plus density-gradient centrifugation for analysis by flow cytometry. CD4^+^ T cells were purified from PBMCs by FITC-conjugated anti-human CD4 mAb and anti-FITC microbeads (Miltenyi Biotec GmbH, DE) according to the manufacturer's instructions.

### 2.3. Flow Cytometric Analysis

For phenotypic analysis, fluorescein isothiocyanate (FITC)-ICOS, phycoerythrin (PE)-CD4, and PE-Cy5-CXCR5 monoclonal antibodies against human-cell surface were purchased from eBioscience (San Diego, CA, USA). All the staining was performed according to the manufacture's protocol. The stained cells were then analyzed using the BD FACScalibur system and CELLQUEST software.

### 2.4. Real-Time Polymerase Chain Reaction

For the detection of IL-21 mRNA of CD4^+^ T cells, CD4^+^ T cells were incubated in complete RPMI1640 culture medium in the presence of 50 ng/mL phorbol myristate acetate (PMA). After 4 h of culture at 37°C under 5% CO_2_, the cells were collected and centrifuged at 500 g for 5 min. After being washed in phosphate-buffered saline (PBS), TRIzol reagent was added. Total RNA was extracted from CD4^+^ T cells with TRIzol reagent and cDNA synthesized according to manufacurer's instructions (Takara, Japan). Each gene was amplified in triplicate, and cDNA concentration differences were normalized to *β*-actin [13]. Primer sequences were as follows: IL-21 sense, 5′-CACAGACTAACATGCCCTTCAT-3′, and antisense, 5′-GAATCTTCACTTC CGTGTGTTCT-3′; Bcl-6 sense, 5′-AAGGCCAGTGAAGCAGAGA-3′, and antisense, 5′-CCGATAGG- CCATGATGTCT-3′.

### 2.5. Enzyme-Linked Immunosorbent Assay (ELISA)

Level of plasma IL-21 was done using ELISA for human IL-21 (eBioscience, San Diego, CA, USA). All determinations were performed by duplicate, and the lower detection limits for IL-21 were 31 pg/mL. The plasma from RA patients was tested for the presence of anticyclic citrullinated peptide (anti-CCP) antibody by ELISA. All samples were analyzed in duplicate using the average of the optical density (OD) values to calculate concentrations.

### 2.6. Statistical Analysis

Data were presented as mean ± standard deviation in the text and figures. Statistical differences were considered to be significant at a value *P* < 0.05 as determined by student's *t*-test using SPSS13.0. Correlation of nonparametric paired data was tested using Spearman's rho, and the significance was evaluated using the t statistic.

## 3. Results

### 3.1. Increased Frequency of the Circulating Tfh Cells in RA Patients

**Figure 1:**
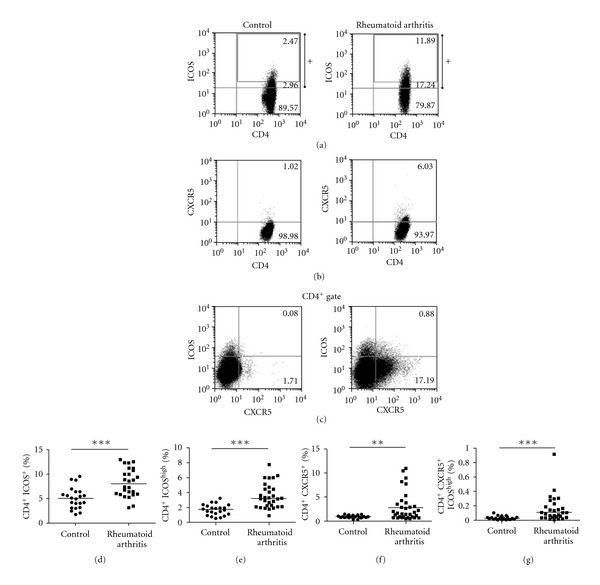
Increased frequency of circulating follicular helper T (Tfh) cells in peripheral blood from rheumatoid arthritis (RA) patients. Peripheral blood mononuclear cells (PMBCs) from RA patients (*n* = 31) and healthy controls (*n* = 30) were stained with labelled antibodies as described in Methods. (a) Representative expression of ICOS versus CD4 expression by flow cytometry, values in each CD4^+^ gate (ICOS^high^ [black box], ICOS positive [ICOS intermediate plus ICOS^high^ [+]], and ICOS negative). (b) Representative flow cytometry plots of Tfh (CD4^+^CXCR5^+^) cells. (c) Representative flow cytometry plots of Tfh (CD4^+^CXCR5^+^ICOS^high^) cells. (d) Percentage of CD4^+^ICOS^+^ T lymphocytes in RA patients and healthy controls (***P < 0.001). (e) Percentage of CD4^+^ICOS^high^ T lymphocytes in RA patients and healthy controls (***P < 0.001). (f) Percentage of CD4^+^CXCR5^+^ T lymphocytes in RA patients and healthy controls (**P < 0.01). (g) Percentage of CD4^+^CXCR5^+^ICOS^high^ T lymphocytes in RA patients and healthy controls (***P < 0.001). Each data point represents an individual subject, and horizontal lines show the median.

Because the inducible costimulatory molecule (ICOS) is a T-cell activation marker, and it has been reported that the frequency of CD4^+^ICOS^+^T cells was increased in RA patients. We first analyzed the frequency of CD4^+^ICOS^+^ and CD4^+^ICOS^high^ T cells in PBMCs from RA patients and healthy controls by flow cytometry. Figures 1(d) and 1(e) showed that the frequencies of CD4^+^ICOS^+^ and CD4^+^ICOS^high^ T cells in RA patients were higher than those in healthy controls (*P* < 0.001). CXCR5 has enabled the Tfh cells to enter the follicles during T-cell-dependent immune responses. In both mice and humans, there have been shown that CXCR5^+^and ICOS^high^ are important phenotype for CD4^+^Tfh cells. We use flow cytometry to analyze the frequency of the circulating Tfh cells in PBMCs from RA patients and healthy controls. A significantly increased frequency of CD4^+^CXCR5^+^ and CD4^+^CXCR5^+^ICOS^high^ circulating Tfh cells was detected in RA patients, compared with that in healthy controls (Figures 1(f) and 1(g)) (*P* < 0.01 and *P* < 0.001, resp.). Our data indicated that increased fraction of the circulating Tfh cells is apparent in a subset of RA patients.

### 3.2. High Levels of Autoantibodies and Increased Frequency of the Circulating Tfh Cells in RA Patients

**Figure 2:**
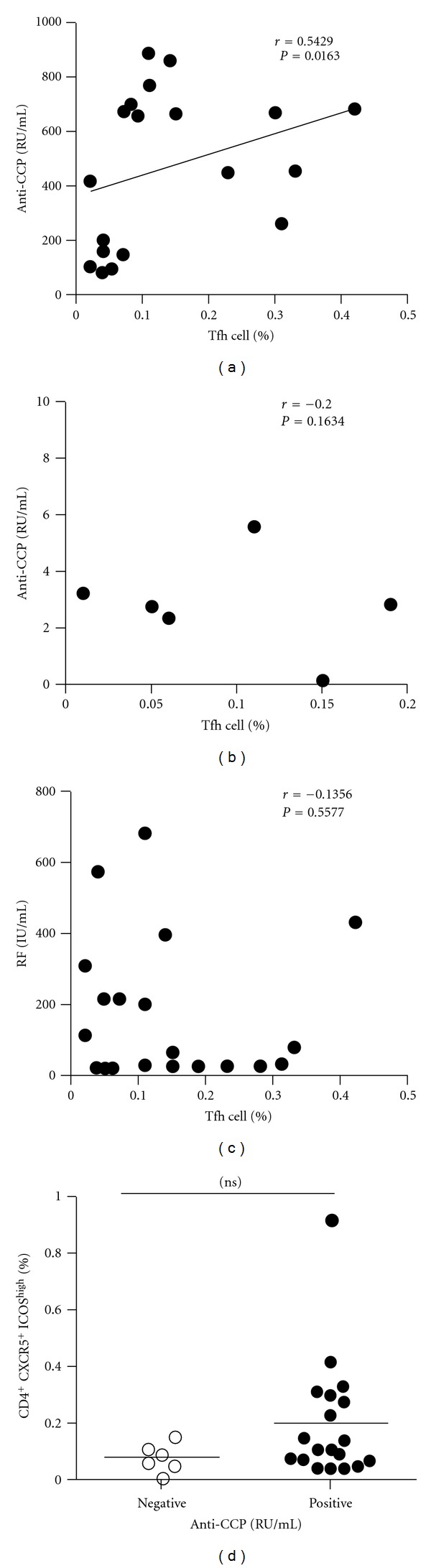
Correlation of circulating Tfh cells frequency and autoantibody in RA patients. (a) Relationship between the frequency of circulating Tfh cells and the positive anti-CCP antibody (*r* = 0.5429, *P* = 0.0163) (*n* = 19). (b) Relationship between the frequency of circulating Tfh cells and the negative anti-CCP antibody (*r* = −0.2000,  *P* = 0.6134) (*n* = 6). (c) Relationship between the frequency of circulating Tfh cells and the level of RF (*r* = −0.1356, *P* = 0.5577) (*n* = 21). (d) Increased frequency of circulating Tfh cells in RA patients according to anti-CCP antibody. Horizontal lines show the median.

In order to analyze the association between autoantibody and the circulating Tfh cells, we compared the levels of plasma autoantibodies (anti-CCP antibody and RF) and frequency of the circulating Tfh in RA patients. According to the titer of anti-CCP antibody, RA patients were divided into two groups: anti-CCP antibody-positive group and anti-CCP antibody-negative group. The frequency of the circulating Tfh cells showed a positive correlation with anti-CCP antibody-positive group in plasma (*r* = 0.5429, *P* = 0.0163, Figure 2(a)) but not with anti-CCP antibody-negative group (*r* = −0.2000, *P* = 0.6134, Figure 2(b)), whereas RF concentrations did not show any correlation with the percentage of the circulating Tfh cells (*r* = −0.1356, *P* = 0.5577, Figure 2(c)). The difference was accounted for by a 3–9-fold increase in CD4^+^CXCR5^+^ICOS^high^ circulating Tfh cells in 7of the 19 RA patients between anti-CCP antibody-positive group and anti-CCP antibody-negative group (Figure 2(d)).

### 3.3. Increased Bcl-6 mRNA, IL-21 mRNA Expression, and IL-21 Concentrations in RA Patients

**Figure 3:**
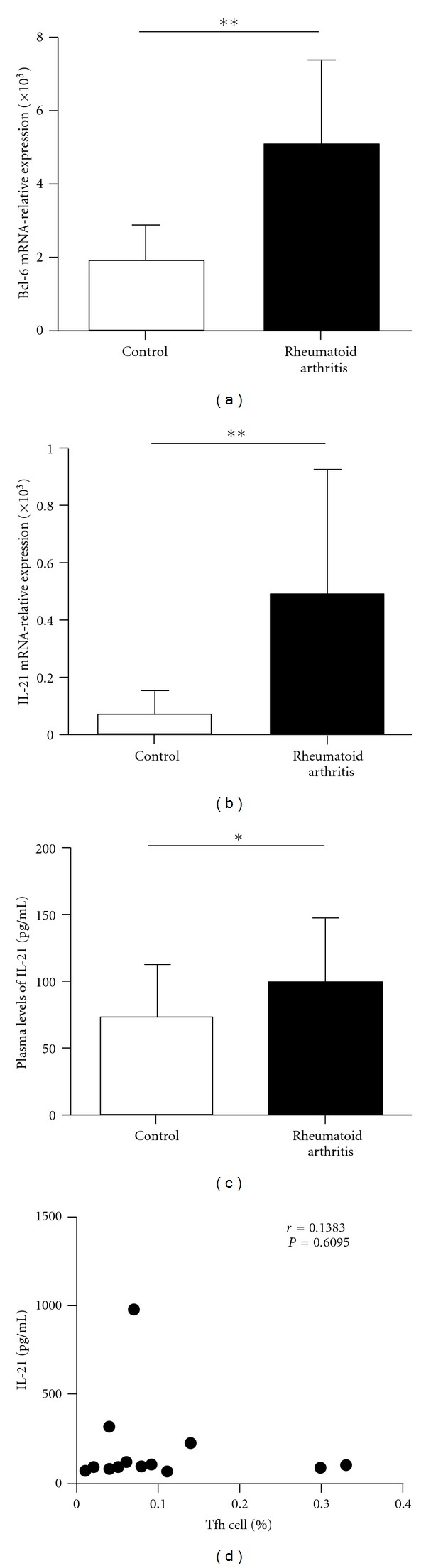
Increased Bcl-6 mRNA, IL-21 mRNA expression, and plasma IL-21 concentration in RA patients. (a) Detection of Bcl-6 mRNA expression in RA patients and healthy controls. Bcl-6 mRNA was estimated by real-time RT-PCR. Data correspond to the mean ± SD of 7 RA patients and 7 healthy controls (**P < 0.01). (b) Detection of IL-21 mRNA expression in RA patients and healthy controls. IL-21 mRNA was estimated by real-time RT-PCR. Data correspond to the mean ± SD of 7 RA patients and 7 healthy controls (**P < 0.01). (c) Plasma IL-21 concentration in RA patients (*n* = 35) and healthy controls (*n* = 29) (*P < 0.05). (d) Relationship between plasma IL-21 concentration and the frequency of circulating Tfh cells in RA patients (*r* = −0.0320, *P* = 0.9100) (*n* = 15).

Previous studies demonstrated that the Bcl-6 was a key transcription factor for Tfh cells. We assessed the expression of transcription factor Bcl-6 in RA patients and healthy controls. Real-time polymerase chain reaction showed that Bcl-6 mRNA expression in CD4^+^ T cells of RA patients was higher than that in control (1.90 ± 1.08 versus 5.24 ± 2.79, *P* < 0.01) (Figure 3(a)). IL-21 is derived from activated CD4^+^ T cells including Tfh cells and could enhance B cells to produce antibody. There was great increased IL-21 mRNA in CD4^+^ T cells of RA patients compared with healthy controls (0.07 ± 0.08 versus 0.48 ± 0.54, *P* < 0.01) (Figure 3(b)). At same time, we found that the concentration of IL-21 in plasma was significant increased in RA patients (98.29 ± 8.40 versus 73.68 ± 7.28, *P* < 0.05) (Figure 3(c)). Additionally, the IL-21 concentrations did not vary with the frequency of the circulating Tfh cells in RA patients (*r* = −0.0321, *P* = 0.9100) (Figure 3(d)).

## 4. Discussion

Tfh cells are a class of regulatory T help cells that specialize in the cognate control of antigen-specific B-cell immunity [7], different from other effector T help cells in function. Tfh cells express CXCR5, CXCR4, ICOS, PD-1, IL-21, and other molecules [14–16]. Notably, highest amounts of ICOS expressed in Tfh cells have a close correlation with the capacity to support antibody production [17, 18]. It has been reported that phenotype of CD4^+^CXCR5^+^ICOS^high^ T cells resembles circulating Tfh cells and tonsillar Tfh cells [11]. In this study, we have investigated the frequency of the circulating Tfh cells in RA patients. The results demonstrate that the frequency of CD4^+^CXCR5^+^ICOS^high^ Tfh cells was increased significantly in RA patients, compared with healthy controls. The percentage of CD4^+^ICOS^+^ cells and CD4^+^ICOS^high^ cells in RA patients were also higher than those in healthy controls. Our data provide first evidence that the circulating Tfh cells were increased in RA patients, suggesting that Tfh cells may be involved in the pathogenesis of RA.

It is known that Tfh cells are critical for directing the development of an antibody response by GC B cell. The presence of autoantibody in the serum is a typical phenomenon for RA patients. Rheumatoid factor (RF) was observed originally by Franklin et al. in 1939 [19], which is present in most inflammatory conditions. Anti-CCP antibody is very specific for RA and has a sensitivity comparable to that of RF [20–22]. We are interested in the relationship between the circulating Tfh cells and autoantibodies in RA patients, including anti-CCP antibody and RF. The results revealed that the increased level of anti-CCP antibody correlated positively with the frequency of circulating Tfh cells, but changes in RF concentrations did not show a correlation with the frequency of circulating Tfh cells. Our data provide a strong association between increased the circulating Tfh cells and RA, which opens a new avenue in the study of RA. As anti-CCP as a kind of representative autoantibody, is not existed in all RA patients. According to the level of anti-CCP antibody, RA patients were divided into two groups: anti-CCP antibody-positive group and anti-CCP antibody-negative group. The results showed that the frequency of circulating Tfh cells in anti-CCP antibody-positive group was higher than that in the anti-CCP antibody-negative group. Taken together, these findings reveal that the production of autoantibody in the RA patients is correlated with the frequency of the circulating Tfh cells, which provide evidence for the link between anti-CCP antibody and the circulating Tfh cells and open a new avenue in the study of RA.

Bryant et al. [23] have found that IL-21 induced the secretion of vast quantities of IgM, IgG, and IgA by all subsets of mature human B cells. Previous study has revealed that IL-21 plasma levels were increased in patients with early stage RA compared with controls [24]. We also detected the concentration of plasma IL-21 and IL-21 mRNA in PBMCs, the results showed that both the levels of IL-21 in plasma and the IL-21 mRNA in CD4^+^ T cells of RA patients were increased significantly. Bcl-6 transcription factor is selectively expressed by Tfh cells [14, 25]. The expression of Bcl-6, regulated by IL-6 and IL-21, helps to distinguish Tfh cells from other polarized T helper cell subsets [26]. Our data indicates that Bcl-6 mRNA markedly enhanced when compared with healthy controls, which was measured in CD4^+^ T cells of RA patients by RT-PCR.

In conclusion, our data provide evidence of increased circulating Tfh cells in RA patients, suggesting that the increase of the circulating Tfh cells may be associated with the development of RA. However, it is critical to further determine the mechanisms of controlling the production and activation of Tfh cells.

## Figures and Tables

**Table 1:** Clinical features of RA patients included in the study.

	RA	Range
*n*	53	
Gender (M/F)	9/44	
Age (yr)	54.44 ± 14.69	
RF (IU/mL)	243.66 ± 427.16	<20 IU/mL
CCP-Ab (RU/mL)	256.81 ± 300.34	<25 RU/mL

Data correspond to the arithmetic mean ± SD, M: Male; F: Female.
